# Timing of Endotracheal Intubation After High-Flow Nasal Cannula Initiation in Patients With Mild-to-Moderate Acute Respiratory Distress Syndrome: A Prospective Observational Cohort Study

**DOI:** 10.7759/cureus.115753

**Published:** 2026-09-03

**Authors:** Niki Pavlatou, Maria Polikandrioti, Evgenia Stergiopoulou, Georgia Toulia, Antonia Kalogianni

**Affiliations:** 1 Department of Nursing, University of West Attica, Athens, GRC; 2 Department of Nursing, Applied Clinical Nursing Program, University of West Attica, Athens, GRC

**Keywords:** acute respiratory distress syndrome, clinical outcomes, endotracheal intubation, high-flow nasal cannula, intensive care

## Abstract

Background: High-flow nasal cannula (HFNC) is widely used in acute hypoxemic respiratory failure, including mild-to-moderate acute respiratory distress syndrome (ARDS). Some patients deteriorate and require invasive mechanical ventilation, but the appropriate timing of intubation remains uncertain. This study examined the association between intubation within 24 hours of HFNC initiation and clinical outcomes among patients with mild-to-moderate ARDS in whom HFNC failed.

Methods: This prospective observational cohort included 109 adults with mild-to-moderate ARDS treated at the General Hospital of Athens "G. Gennimatas," Greece, from August 2023 to August 2024. All initially received HFNC and were subsequently intubated. Mild and moderate ARDS were classified by Berlin oxygenation criteria (PaO₂/FiO₂ 201-300 and 101-200 mmHg, respectively). HFNC failure was defined as subsequent endotracheal intubation and invasive mechanical ventilation. A predefined 24-hour cutoff, supported by previous studies, classified patients as early or delayed intubation. Intubation decisions were based on arterial blood gases and overall clinical condition. Outcomes included in-hospital mortality, successful ventilator weaning, hospital and intensive care unit (ICU) length of stay, and duration of the initial controlled mechanical ventilation phase. Multivariable models adjusted for age, sex, body mass index, comorbidity burden, admission blood pressure, and peripheral oxygen saturation.

Results: Twenty-two patients (20.2%) were intubated within 24 hours and 87 (79.8%) after 24 hours. In-hospital death occurred in 0 of 22 patients (0.0%) in the early group and 15 of 87 (17.2%) in the delayed group (p = 0.038). Successful ventilator weaning occurred in 17 of 22 patients (77.3%) and six of 87 (6.9%), respectively (p < 0.001). After adjustment, intubation within 24 hours was associated with lower odds of in-hospital death (OR = 0.03, 95% CI: 0.001-0.89; p = 0.043), although the wide confidence interval indicates substantial uncertainty. The early group had a longer hospital stay (adjusted β = 0.14, 95% CI: 0.03-0.25; p = 0.017), shorter ICU stay (adjusted β = −0.44, 95% CI: −0.72 to −0.17; p = 0.002), and shorter initial controlled mechanical ventilation phase (adjusted β = −0.30, 95% CI: −0.53 to −0.07; p = 0.015).

Conclusion: Among patients with mild-to-moderate ARDS requiring invasive mechanical ventilation after HFNC failure, intubation within 24 hours was associated with lower observed in-hospital mortality, more frequent successful ventilator weaning, and shorter ICU and controlled ventilation durations. These findings support close monitoring and timely escalation when deterioration indicates insufficient non-invasive support. The observational design and limited outcome events preclude causal interpretation, and larger multicenter cohorts are needed to confirm these associations.

## Introduction

Acute respiratory distress syndrome (ARDS) is an acute inflammatory lung injury characterized by increased pulmonary vascular permeability, loss of aerated lung tissue, bilateral pulmonary opacities, and hypoxemic respiratory failure [1]. The Berlin definition classifies ARDS severity according to the ratio of arterial oxygen tension to the fraction of inspired oxygen (PaO₂/FiO₂) [1,2]. The more recent Global Definition of ARDS extends the diagnostic framework to nonintubated patients receiving high-flow nasal oxygen at flow rates of at least 30 L/min [3]. Despite advances in respiratory support and critical care, ARDS remains associated with substantial morbidity and mortality, particularly among patients who progress to invasive mechanical ventilation [4].

High-flow nasal cannula (HFNC) therapy is widely used in acute hypoxemic respiratory failure, including patients with mild-to-moderate ARDS. HFNC delivers heated and humidified oxygen at high flow rates, improves oxygen delivery, reduces anatomical dead space, and may decrease inspiratory effort [5,6]. Clinical studies have shown that HFNC can improve patient comfort and increase ventilator-free days in selected patients with acute hypoxemic respiratory failure [7]. Current clinical guidance supports its use in appropriately monitored patients while emphasizing the need for timely recognition of treatment failure [8,9].

A proportion of patients deteriorate despite HFNC therapy and subsequently require endotracheal intubation and invasive mechanical ventilation. Determining the appropriate time for intubation remains difficult because early invasive ventilation exposes patients to risks associated with sedation, ventilator-associated pneumonia, and ventilator-induced lung injury [10]. Continued HFNC support during progressive respiratory failure may prolong excessive spontaneous inspiratory effort, increase transpulmonary pressure fluctuations, and contribute to patient self-inflicted lung injury [11,12]. Clinical deterioration may also reflect progression of the underlying inflammatory disease rather than worsening gas exchange alone. Interleukin-6 (IL-6) is an important mediator of systemic inflammatory responses, and elevated IL-6 levels have been associated with greater disease severity and adverse prognosis in severe inflammatory conditions, including COVID-19 [13]. These competing risks and mechanisms require repeated clinical assessment rather than reliance on a single physiological variable.

Previous observational studies have reported an association between delayed intubation after HFNC treatment and increased mortality [14]. However, the available evidence remains difficult to interpret because intubation timing is influenced by baseline disease severity, clinical response, physician judgment, treatment location, and access to intensive care resources [15-17]. Differences in the definition of HFNC failure and in the time point used to classify early and delayed intubation further limit comparisons across studies.

The objective of this study was to evaluate the association between intubation timing, within 24 hours or after 24 hours of HFNC initiation, and clinical outcomes among patients with mild-to-moderate ARDS in whom HFNC therapy failed and invasive mechanical ventilation became necessary. The evaluated outcomes included in-hospital mortality, successful ventilator weaning, hospital length of stay, intensive care unit (ICU) length of stay, and the duration of the initial controlled mechanical ventilation phase.

## Materials and methods

Study design and setting

This was a single-center prospective observational cohort study designed to investigate the association between the timing of endotracheal intubation following initiation of HFNC therapy and clinical outcomes in adults with mild-to-moderate ARDS. Consecutive patients receiving HFNC therapy were prospectively enrolled, and only patients in whom HFNC therapy failed and invasive mechanical ventilation became necessary were included in the final analysis. The study was conducted in the medical wards and ICU of the General Hospital of Athens "G. Gennimatas," Athens, Greece, between August 2023 and August 2024.

The initiation of HFNC therapy and the decision to proceed with endotracheal intubation were determined by the attending clinical team as part of routine clinical practice and were not assigned by the study protocol. Immediate transfer to the ICU after intubation depended on ICU bed availability. When an ICU bed was unavailable, patients continued to receive invasive mechanical ventilation in the medical wards until transfer to the ICU became possible.

The study is reported in accordance with the Strengthening the Reporting of Observational Studies in Epidemiology (STROBE) recommendations for cohort studies [18].

Inclusion and exclusion criteria

Patients were eligible for the prospective cohort if they were aged 18 years or older, had acute hypoxemic respiratory failure fulfilling the clinical and imaging features of ARDS, and received HFNC therapy. In the revised diagnostic framework, HFNC treatment at flow rates of at least 30 L/min is consistent with the nonintubated ARDS category of the global definition [3]. For the present analysis, only patients who subsequently required endotracheal intubation and invasive mechanical ventilation were included.

Patients were excluded from the prospective cohort if they were younger than 18 years, were pregnant, had severe ARDS, or had incomplete clinical data required for the planned analyses. Patients who responded successfully to HFNC therapy and did not require endotracheal intubation were excluded from the final analysis.

Participants

Patients were recruited consecutively from the medical wards and ICU of the General Hospital of Athens "G. Gennimatas," Athens, Greece, between August 2023 and August 2024. During the study period, 179 patients receiving HFNC therapy for acute hypoxemic respiratory failure were assessed for eligibility. Thirty-two of 179 patients (17.9%) were excluded: six patients (3.4%) were younger than 18 years, 12 patients (6.7%) had severe ARDS according to the Berlin definition, two patients (1.1%) were pregnant, and 12 patients (6.7%) had incomplete clinical data required for the planned analyses.

After applying the eligibility criteria, 147 of 179 patients (82.1%) were included in the prospective cohort. Of these, 38 of 147 patients (25.9%) responded successfully to HFNC therapy and did not require endotracheal intubation during hospitalization. These patients were excluded from the final analysis because the study question concerned the timing of endotracheal intubation among patients in whom HFNC ultimately failed and invasive mechanical ventilation became necessary. Patients who did not require intubation could not be classified according to the predefined exposure of intubation within or after 24 hours and therefore were outside the analytic population for this comparison.

The final study population therefore comprised 109 of 147 eligible patients (74.1%) who subsequently underwent endotracheal intubation and invasive mechanical ventilation. Patients were classified according to the interval between HFNC initiation and endotracheal intubation. A 24-hour cutoff was predefined in the study protocol and was supported by previous studies evaluating intubation timing after HFNC therapy [14-17]. Twenty-two of 109 patients (20.2%) were intubated within 24 hours of HFNC initiation and were assigned to the early-intubation group, whereas 87 of 109 patients (79.8%) were intubated after 24 hours and were assigned to the delayed-intubation group. The patient selection process is presented in Figure 1.

**Figure 1:**
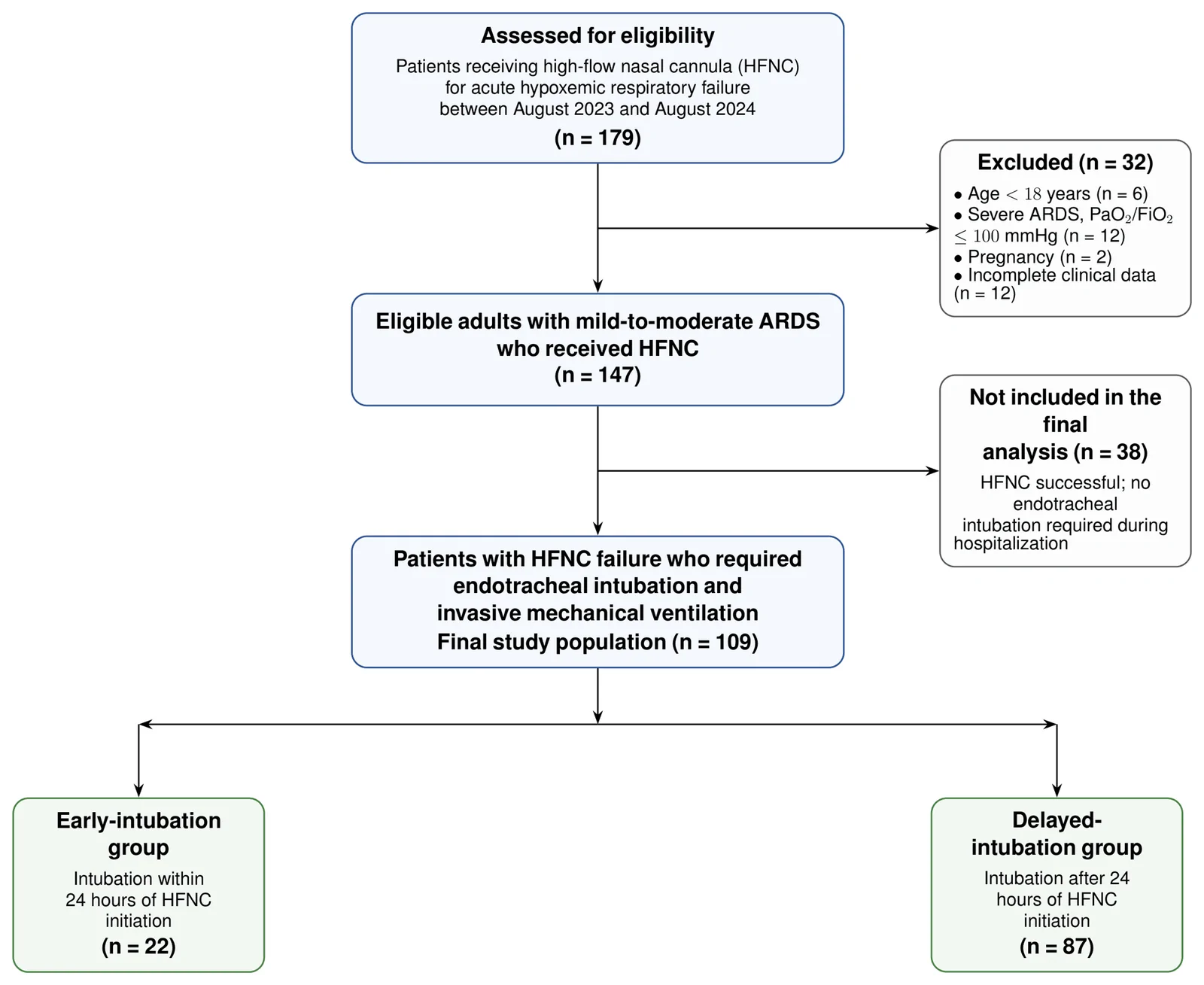
Flow diagram of patient selection ARDS: acute respiratory distress syndrome; HFNC: high-flow nasal cannula

Study protocol

At enrolment, demographic and lifestyle characteristics were recorded, including age, sex, marital status, educational level, body mass index, smoking status, and physical activity. Medical history was reviewed for cardiovascular, endocrine, respiratory, neurological, peptic ulcer, liver, chronic kidney, malignant, and infectious diseases. The total number of comorbidities was calculated for each patient.

HFNC therapy was administered using the AIRVO™ 2 system (Fisher & Paykel Healthcare, Auckland, New Zealand) according to the hospital protocol. Initial flow rates ranged from 40 to 60 L/min, and the gas temperature was maintained at 37°C. The fraction of inspired oxygen (FiO₂) was adjusted to maintain peripheral oxygen saturation (SpO₂) between 92% and 96%.

Initial HFNC settings were individualized according to the patient's oxygenation status, respiratory effort, and the treating physician's clinical judgment.

Clinical and laboratory variables were recorded at admission, at the 24-hour assessment, and on the first day after intubation, where applicable. These time points refer to standardized study data collection; patients remained under continuous clinical monitoring during HFNC therapy. Clinical measurements included systolic, diastolic, and mean arterial pressure, heart rate, body temperature, respiratory rate, and SpO₂. Arterial blood gas measurements included pH, arterial oxygen tension (PaO₂), arterial carbon dioxide tension (PaCO₂), bicarbonate (HCO₃⁻), and lactate. Additional laboratory measurements included sodium, magnesium, potassium, hemoglobin, hematocrit, platelet count, white blood cell count, C-reactive protein, albumin, urea, creatinine, and glucose. Major in-hospital complications were also recorded during hospitalization, including respiratory infections, pneumonia, and sepsis.

Standardized critical care severity scores, including Acute Physiology and Chronic Health Evaluation II (APACHE II), Simplified Acute Physiology Score II (SAPS II), and Sequential Organ Failure Assessment (SOFA), were not prospectively recorded. Detailed ventilator mechanics after intubation, including PEEP and plateau pressure, were also not available for analysis.

ARDS was diagnosed by the attending physicians based on the clinical presentation, chest imaging findings, and oxygenation criteria. Patients received HFNC at flow rates of 40-60 L/min, which satisfies the respiratory-support threshold of at least 30 L/min included in the global definition for nonintubated ARDS [3]. For descriptive oxygenation stratification, a PaO₂/FiO₂ ratio of 201-300 mmHg was classified as mild, and a ratio of 101-200 mmHg as moderate, corresponding to the established Berlin oxygenation ranges [1].

Criteria for endotracheal intubation

Patients were continuously monitored throughout HFNC therapy. The decision to proceed with endotracheal intubation was made by the attending intensivists and pulmonologists as part of routine clinical care, based on arterial blood gas findings and the patient's overall clinical condition.

Intubation was considered in the presence of persistent or worsening hypoxemia, deterioration in the PaO₂/FiO₂ ratio, increased work of breathing or respiratory distress, respiratory acidosis or hypercapnia, hemodynamic instability, or neurological deterioration that impaired airway protection. Work of breathing was assessed clinically, including the use of accessory respiratory muscles, together with the patient's oxygenation status. The respiratory rate-oxygenation (ROX) index was not prospectively recorded or used to guide the decision to intubate. No predefined PaO₂/FiO₂ threshold was used as the sole indication for intubation. The final decision was individualized according to the patient's clinical course and response to HFNC therapy.

The decision to intubate was based on the overall clinical assessment rather than on any isolated physiological parameter.

Definition of HFNC failure

For the purposes of cohort eligibility, HFNC failure was defined as the subsequent requirement for endotracheal intubation and initiation of invasive mechanical ventilation after HFNC therapy.

Time to intubation was calculated from the initiation of HFNC therapy to endotracheal intubation. Patients intubated within 24 hours of HFNC initiation were classified in the early-intubation group, whereas those intubated after 24 hours were classified in the delayed-intubation group.

Outcome definitions

The study outcomes evaluated were in-hospital mortality, successful ventilator weaning, immediate admission to the ICU after intubation, hospital length of stay, ICU length of stay, duration of the initial controlled mechanical ventilation phase, complications, and death during the ICU stay.

Death during the ICU stay was defined as death occurring at any time during ICU hospitalization among patients who were admitted to the ICU, irrespective of whether ICU admission occurred immediately after intubation or after a delay due to ICU bed availability.

In-hospital mortality was defined as death from any cause before discharge from the index hospitalization. Successful ventilator weaning was defined as sustained spontaneous breathing following extubation without reintubation or renewed invasive mechanical ventilation during the subsequent 48 hours.

The duration of the initial controlled mechanical ventilation phase was defined as the interval, expressed in days, from the initiation of invasive mechanical ventilation in volume-controlled or pressure-controlled mode to the first transition to an assisted ventilation mode, such as pressure-support ventilation.

Immediate admission to the ICU after intubation was defined as direct transfer to the ICU when a bed was available. Patients who could not be transferred immediately continued to receive invasive mechanical ventilation in the medical wards until an ICU bed became available. This variable was considered a descriptor of the care pathway rather than an independent clinical outcome.

Hospital length of stay was recorded in days for all included patients. ICU length of stay was recorded only for patients who were admitted to the ICU.

Complications were defined as the occurrence of major in-hospital clinical complications during hospitalization, most commonly respiratory infections, pneumonia, and sepsis.

Ethical considerations

The study was conducted in accordance with the principles of the Declaration of Helsinki. Ethical approval was obtained from the Research Ethics and Deontology Committee of the University of West Attica, Athens, Greece (approval number 71318/25-07-2023; approval date July 25, 2023).

Written informed consent was obtained from all participants or, when applicable, from their legally authorized representatives before enrolment. Participants were informed of the study purpose, the voluntary nature of participation, the confidentiality of their data, and their right to withdraw without affecting their clinical care.

Statistical analysis

Continuous variables were summarized as mean values with standard deviations or as medians with interquartile ranges, according to their distribution. Categorical variables were summarized as absolute frequencies and percentages. Comparisons between the early- and delayed-intubation groups were performed using Pearson's chi-square test, the independent-samples Student's t-test, or the Mann-Whitney U test, as appropriate.

Outcomes that differed significantly between the two groups in the unadjusted analyses were subsequently examined as dependent variables in linear or logistic regression models, according to outcome type. The multivariable models were adjusted for age, sex, body mass index, number of comorbidities, systolic blood pressure, diastolic blood pressure, and peripheral oxygen saturation at admission. These covariates represented baseline demographic characteristics, body habitus, comorbidity burden, hemodynamic status, and oxygenation. Patients with incomplete clinical data required for the planned analyses had been excluded during cohort assembly. Logistic regression results were expressed as odds ratios with 95% confidence intervals, whereas linear regression results were expressed as adjusted regression coefficients with 95% confidence intervals.

The assumptions of linear regression were assessed before model interpretation. Logarithmic transformations were applied to non-normally distributed dependent variables when required to satisfy these assumptions. Firth-penalized logistic regression was used for outcomes with zero events or complete separation [19,20], for which conventional maximum likelihood estimation may produce unstable or non-estimable coefficients. Given the limited number of outcome events, adjusted estimates with wide confidence intervals were interpreted cautiously. All statistical tests were two-sided, and statistical significance was set at p < 0.05. Analyses were conducted using Statistical Product and Service Solutions (SPSS, version 27.0; IBM SPSS Statistics for Windows, Armonk, NY).

## Results

The study included 109 patients who received HFNC therapy and subsequently underwent endotracheal intubation and invasive mechanical ventilation. The mean age was 72.5 years (standard deviation (SD) = 11.2 years), and 58 patients (53.2%) were men. Twenty-two patients (20.2%) were intubated within 24 hours of HFNC initiation, whereas 87 patients (79.8%) were intubated after 24 hours.

Baseline characteristics of the study population according to intubation timing are presented in Table 1. Categorical baseline characteristics of the study population according to intubation timing are presented in Table 2. Age, sex, body mass index, educational level, smoking status, and physical activity did not differ significantly between the early- and delayed-intubation groups.

**Table 1:** Baseline characteristics of the study population according to intubation timing Data are presented as mean ± SD. BMI: body mass index

Parameters	Total sample (n=109)	Intubation after 24 h (n=87)	Intubation within 24 h (n=22)	t (df)
Age, years	72.5 ± 11.2	72.5 ± 11.5	72.4 ± 10.1	-0.05 (107)
BMI, kg/m^2^	29.6 ± 6.1	29.2 ± 6.0	31.2 ± 6.4	1.40 (107)

**Table 2:** Categorical baseline characteristics of the study population according to intubation timing Data are presented as n (%). BMI: body mass index

Parameters	Alternatives	Total sample (n=109)	Intubation during the 1^st^ day		^χ2^ (df)
			No (n=87; 79.8%)	Yes (n=22; 20.2%)	
		n (%)	n (%)	n (%)	
Gender	Men	58 (53.2)	45 (51.7)	13 (59.1)	0.38 (1)
	Women	51 (46.8)	42 (48.3)	9 (40.9)	
Marital status	Married	55 (50.5)	45 (51.7)	10 (45.5)	0.28 (1)
	Not married	54 (49.5)	42 (48.3)	12 (54.5)	
BMI levels	Normal	22 (20.2)	19 (21.8)	3 (13.6)	3.79 (2)
	Overweight	47 (43.1)	40 (46.0)	7 (31.8)	
	Obese	40 (36.7)	28 (32.2)	12 (54.5)	
Educational level	Primary	24 (22.0)	19 (21.8)	5 (22.7)	0.61 (2)
	Secondary	66 (60.6)	54 (62.1)	12 (54.5)	
	University	19 (17.4)	14 (16.1)	5 (22.7)	
Smoking status	Smoking	61 (56.0)	48 (55.2)	13 (59.1)	0.11 (1)
	Non-smoking	48 (44.0)	39 (44.8)	9 (40.9)	
Physical activity	Not at all	102 (93.6)	81 (93.1)	21 (95.5)	0.16 (1)
	Once a week	7 (6.4)	6 (6.9)	1 (4.5)	

Comorbidities are presented in Table 3. Cardiovascular disease was the most frequent comorbidity, affecting 88 (80.7%) patients, followed by respiratory disease in 81 (74.3%) patients and endocrine disease in 73 (67.0%) patients. All patients had at least one comorbidity, and the median number of comorbidities was four (interquartile range (IQR) = 3-4). The distribution of individual comorbidities and the total comorbidity burden did not differ significantly between the two groups.

**Table 3:** Comorbidities of the study population according to intubation timing Data are presented as n (%) or median (IQR). CKD: chronic kidney disease

Parameters	Total sample (n=109)	No (n=87; 79.8%)	Yes (n=22; 20.2%)	χ^2^ (df)
	n (%)	n (%)	n (%)	
Cardiovascular disease	88 (80.7)	70 (80.5)	18 (81.8)	0.02 (1)
Endocrine disease	73 (67.0)	55 (63.2)	18 (81.8)	2.75 (1)
Respiratory disease	81 (74.3)	65 (74.7)	16 (72.7)	0.04 (1)
Neurological disease	47 (43.1)	40 (46.0)	7 (31.8)	1.44 (1)
Peptic ulcer	12 (11.0)	11 (12.6)	1 (4.5)	1.18 (1)
Liver disease	6 (5.5)	5 (5.7)	1 (4.5)	0.05 (1)
CKD	10 (9.2)	8 (9.2)	2 (9.1)	0.00 (1)
Cancer	35 (32.1)	25 (28.7)	10 (45.5)	2.25 (1)
Infectious diseases	28 (25.7)	19 (21.8)	9 (40.9)	3.35 (1)
	Median (IQR)	Median (IQR)	Median (IQR)	U
Number of comorbidities	4 (3-4)	4 (2-4)	4 (3-5)	827.5

The clinical and laboratory parameters recorded at admission and after intubation are presented in Table 4. At admission, systolic blood pressure was lower in the early-intubation group than in the delayed-intubation group (U = 695.5, p = 0.048). Serum lactate was higher among patients intubated within 24 hours (U = 553.0, p = 0.002). No statistically significant between-group differences were observed for the remaining clinical, arterial blood gas, or laboratory variables at admission.

**Table 4:** Clinical and laboratory characteristics at admission and on the first day after intubation according to intubation timing Data are presented as median (IQR). Values marked with * and** indicate statistically significant between-group differences at p < 0.05 and p < 0.01, respectively. SBP: systolic blood pressure; DBP: diastolic blood pressure; MBP: mean blood pressure; SpO₂: peripheral oxygen saturation; pH: potential of hydrogen; PaO₂: partial pressure of arterial oxygen; PaCO₂: partial pressure of arterial carbon dioxide; Na: sodium; Mg: magnesium; K⁺: potassium; HCO₃: bicarbonate; Hb: hemoglobin; Hct: hematocrit; WBC: white blood cell count; CRP: C-reactive protein; FiO₂: fraction of inspired oxygen; P/F ratio: PaO₂/FiO₂ ratio

Parameters	Total sample (n=109)	Intubation during the 1^st^ day		U
		No (n=87; 79.8%)	Yes (n=22; 20.2%)	
	Median (IQR)	Median (IQR)	Median (IQR)	
At admission				
SBP	95.9 (24.6)	97.83 (25.06)	88.45 (21.33)	695.5*
DBP	54.1 (13.7)	53.99 (13.11)	54.55 (16.36)	901
MBP	74.4 (18)	75.48 (18.58)	70 (14.79)	751.5
SpO_2_	87.69 (1.71)	87.77 (1.75)	87.36 (1.53)	854
Heart rate	92.2 (17.2)	91.85 (17.33)	93.59 (17.26)	892.5
Temperature (°C)	37.8 (1.2)	37.74 (1.23)	38.06 (0.83)	851
Respiratory frequency	27.3 (3.8)	27.17 (3.76)	27.68 (4)	888.5
pH	7.37 (0.08)	7.37 (0.08)	7.38 (0.06)	931.5
PaO_2_	56.89 (6.82)	57 (7.19)	56.45 (5.19)	955.5
PaCO_2_	43.21 (8.46)	42.62 (8.61)	45.55 (7.6)	734.5
Na	134.04 (7.55)	133.78 (7.24)	135.05 (8.77)	876.5
Mg	2.09 (0.42)	2.09 (0.4)	2.09 (0.52)	840
Potassium (K+)	4.16 (0.75)	4.19 (0.77)	4.03 (0.68)	844
HC0_3_	25.7 (3.21)	25.44 (3.06)	26.71 (3.67)	726
Hb	10.92 (3.06)	10.93 (3.07)	10.87 (3.11)	951.5
Hct	33.85 (9.2)	33.79 (9.04)	34.08 (10.05)	948.5
Platelets	252.73 (103.61)	261.26 (107.06)	219 (82.32)	738.5
WBC	11.99 (5)	11.9 (5.16)	12.34 (4.4)	854
CRP	17.83 (8.34)	17.6 (7.79)	18.74 (10.41)	929.5
Albumin	3.36 (0.61)	3.4 (0.63)	3.19 (0.47)	814.5
Urea	63.08 (28.94)	60.92 (25)	71.64 (40.64)	741.5
Creatinine	1.11 (0.45)	1.08 (0.43)	1.25 (0.49)	777.5
Lactate	2.49 (0.89)	2.35 (0.79)	3.03 (1.07)	553**
Glucose	165.83 (73.47)	163.89 (77.16)	173.55 (57.44)	847.5
On the 1st day of intubation				
FiO_2 _	0.55 (0.16)	0.55 (0.18)	0.55 (0.14)	157.5
P/F ratio	128 (30.3)	131.7 (29.8)	125.1 (31.1)	133.5

On the first day after intubation, the groups had similar FiO₂ requirements and PaO₂/FiO₂ ratios, with no statistically significant between-group differences.

Immediate admission to the ICU after intubation was recorded for all 22 patients in the early-intubation group and for 18 (20.7%) of the 87 patients in the delayed-intubation group (100.0% vs. 20.7%; χ²(1) = 47.55, p < 0.001). This variable reflected immediate ICU bed availability and the corresponding care pathway after intubation. Table 5 presents the clinical outcomes according to intubation timing.

**Table 5:** Clinical outcomes according to intubation timing Data are presented as n (%) or median (IQR). Values marked with *, **, and *** indicate statistically significant between-group differences at p < 0.05, p < 0.01, and p < 0.001, respectively. VC-PC duration: duration of the initial colume-controlled/pressure-controlled mechanical ventilation phase; ICU: intensive care unit

Parameters	Total sample (n=109)	Intubation during the 1^st^ day		χ^2^ (df)
		No (n=87; 79.8%)	Yes (n=22; 20.2%)	
	n (%)	n (%)	n (%)	
Immediate ICU admission after intubation	40 (36.7)	18 (20.7)	22 (100)	47.55 (1)***
Successful ventilator weaning	23 (21.1)	6 (6.9)	17 (77.3)	52.24 (1)***
Death	15 (13.8)	15 (17.2)	0 (0.0)	4.40 (1)*
Complications	40 (36.7)	28 (32.2)	12 (54.5)	3.78 (1)
Death in ICU	14 (12.8)	14 (16.1)	0 (0.0)	4.06 (1)
Others	Median (IQR)	Median (IQR)	Median (IQR)	U
Duration of hospitalization (days)	22 (16-32)	19 (14-27)	32 (27-46)	411.5***
ICU length of stay	30 (14-62)	69 (37-84)	16 (11-30)	41.5***
VC-PC duration	14 (12-20)	18 (13-26)	12 (7-17.5)	50*

Successful ventilator weaning occurred in 17 of 22 patients (77.3%) in the early-intubation group and in six of 87 patients (6.9%) in the delayed-intubation group (χ²(1) = 52.24, p < 0.001; Figure 2). In-hospital death occurred in 0 of 22 patients (0.0%) in the early-intubation group and in 15 of 87 patients (17.2%) in the delayed-intubation group (χ²(1) = 4.40, p = 0.038). The frequencies of complications and death during the ICU stay did not differ significantly between the groups.

**Figure 2:**
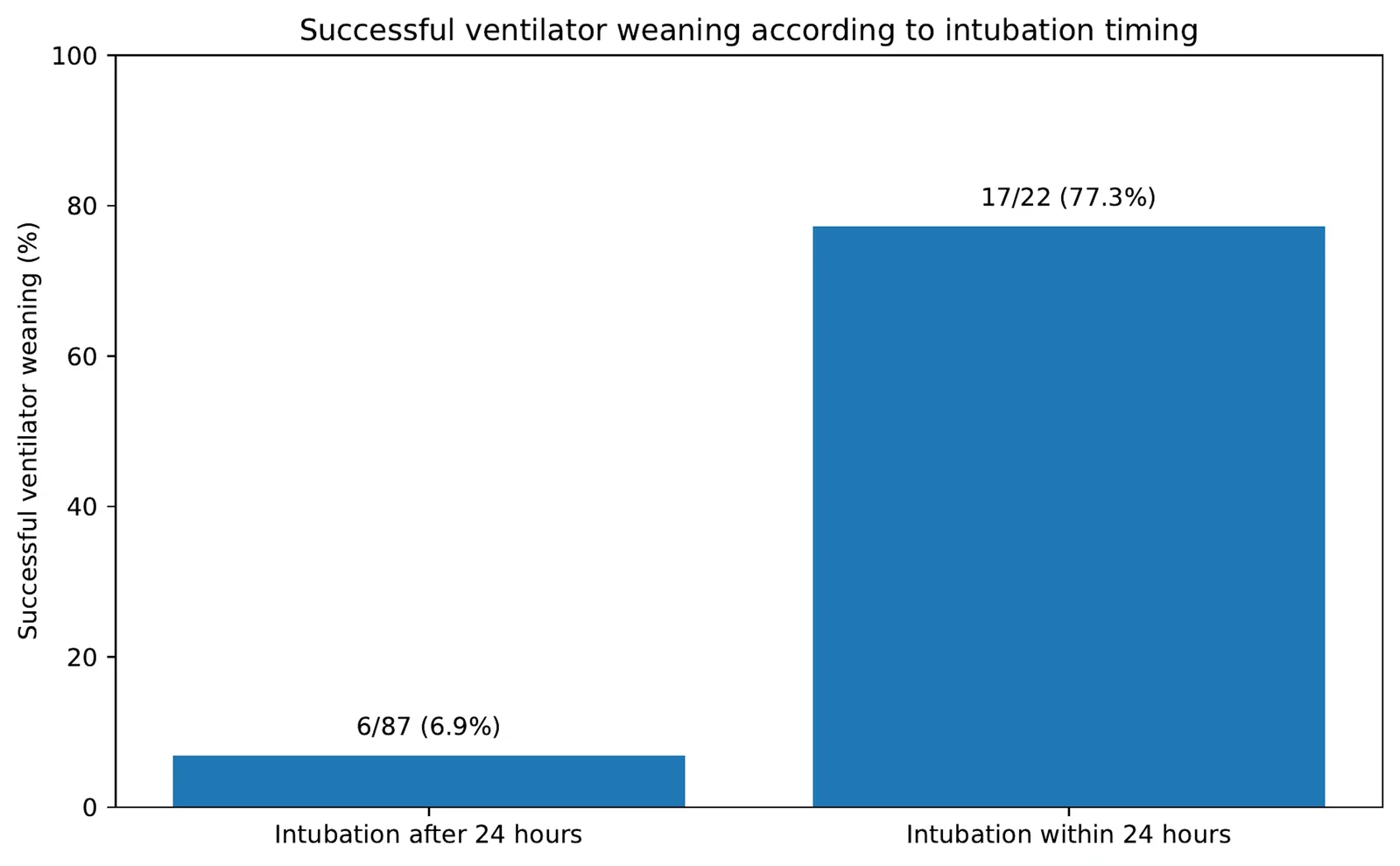
Percentages of successful ventilator weaning

Hospital length of stay was longer in the early-intubation group than in the delayed-intubation group (median: 32 days (IQR: 27-46) vs. 19 days (IQR: 14-27); U = 411.5, p < 0.001). Among patients who were admitted to the ICU, ICU length of stay was shorter in the early-intubation group (median: 16 days (IQR: 11-30) vs. 69 days (IQR: 37-84); U = 41.5, p < 0.001; Figure 3). The duration of the initial controlled mechanical ventilation phase was also shorter among patients intubated within 24 hours (median: 12 days (IQR: 7-17.5) vs. 18 days (IQR: 13-26); U = 50.0, p = 0.018).

**Figure 3:**
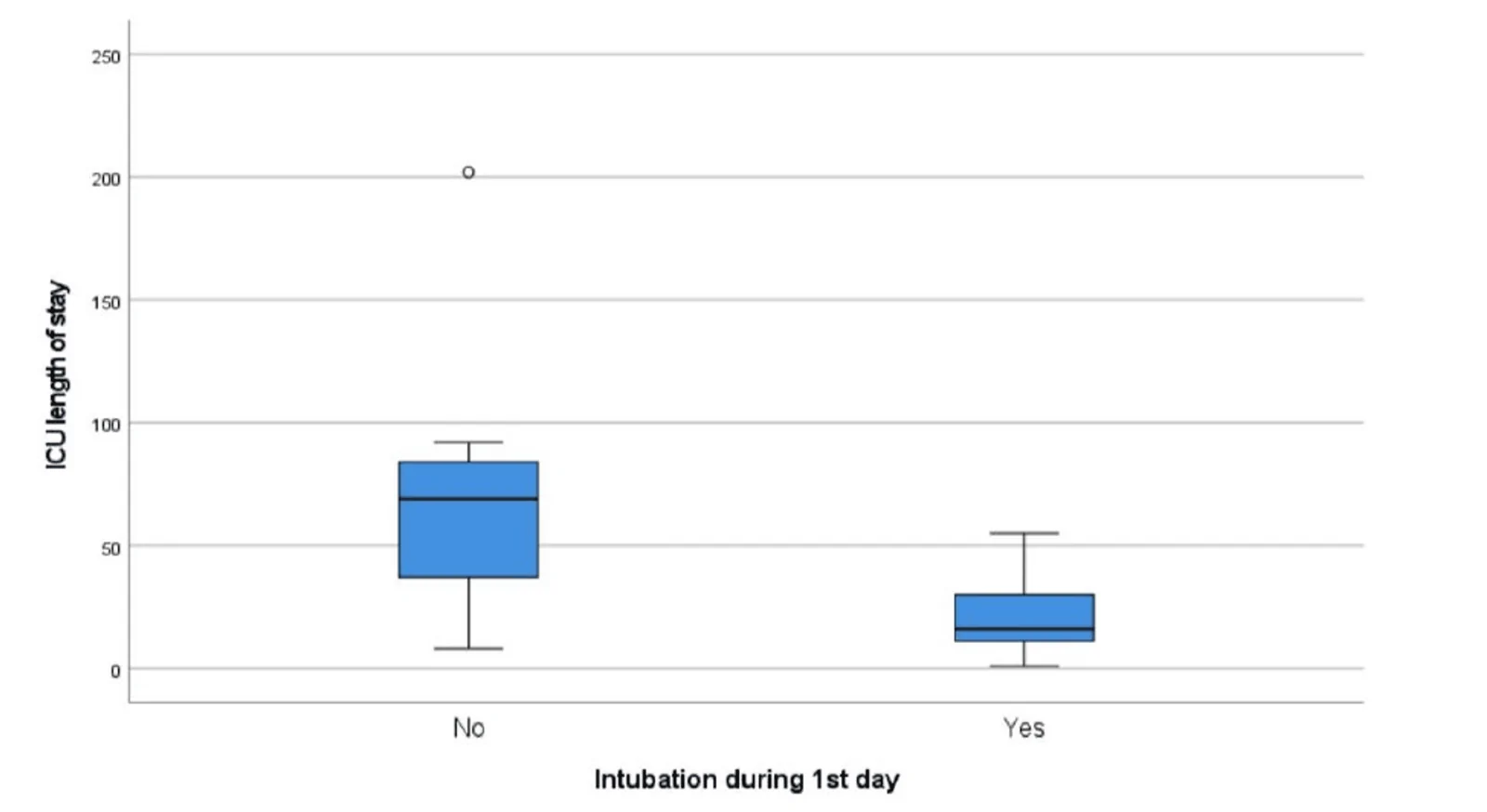
Boxplot of ICU length of stay according to intubation within the first 24 hours after HFNC initiationFirth penalized logistic regression The horizontal line represents the median, the box indicates the interquartile range (IQR), whiskers represent the minimum and maximum values excluding outliers, and open circles (○) denote outlier observations.

Because the three continuous outcomes were logarithmically transformed, the reported adjusted regression coefficients are expressed on the logarithmic scale. After adjustment for age, sex, body mass index, number of comorbidities, systolic blood pressure, diastolic blood pressure, and admission SpO₂, hospital length of stay was longer among patients intubated within 24 hours (adjusted β = 0.14, 95% confidence interval (CI): 0.03-0.25; p = 0.017). ICU length of stay remained shorter in the early-intubation group (adjusted β = −0.44, 95% CI: −0.72 to −0.17; p = 0.002). The duration of the initial controlled mechanical ventilation phase also remained shorter among patients intubated within 24 hours (adjusted β = −0.30, 95% CI: −0.53 to −0.07; p = 0.015).

Firth penalized logistic regression showed that intubation within 24 hours of HFNC initiation was associated with lower odds of in-hospital death after adjustment for the same covariates (odds ratio (OR) =0.03, 95% CI: 0.001-0.89; p = 0.043). The confidence interval was wide, indicating substantial uncertainty in the magnitude of this association. Early intubation was also associated with higher odds of immediate ICU admission after intubation (OR = 45.6, 95% CI: 1.90-107.71; p < 0.001) and successful ventilator weaning (OR = 8.5, 95% CI: 1.43-50.6; p < 0.001).

## Discussion

In this prospective observational cohort of patients with mild-to-moderate ARDS who ultimately required invasive mechanical ventilation, intubation within 24 hours of HFNC initiation was associated with lower observed in-hospital mortality, more frequent successful ventilator weaning, a shorter ICU stay, and a shorter initial controlled mechanical ventilation phase. These associations remained statistically significant after adjustment for age, sex, body mass index, number of comorbidities, admission blood pressure, and peripheral oxygen saturation. Patients intubated within 24 hours had a longer overall hospital stay.

The findings are consistent with previous observational studies reporting less favorable outcomes when intubation is delayed during unsuccessful HFNC treatment [14,21,22]. Direct comparison across studies remains difficult because definitions of HFNC failure, exposure time, patient population, and outcome assessment vary. In the present study, all included patients were eventually intubated, and the 24-hour interval was measured from HFNC initiation. The analysis therefore compares earlier and later intubation within a cohort in which HFNC ultimately failed, rather than comparing intubated and non-intubated patients.

One possible explanation for the observed associations is that prolonged spontaneous breathing during progressive respiratory failure may expose the injured lung to sustained inspiratory effort and large transpulmonary pressure fluctuations. These conditions have been proposed as contributors to patient self-inflicted lung injury and respiratory muscle fatigue [11,12]. The present study did not measure inspiratory effort, esophageal pressure, transpulmonary pressure, or biological markers of lung injury. The results are compatible with this mechanism but do not establish it.

Clinical deterioration during HFNC may also reflect progression of the underlying systemic inflammatory response in addition to worsening respiratory mechanics and gas exchange. IL-6 has an established role in inflammatory signaling, and elevated circulating IL-6 concentrations have been associated with greater disease severity and adverse prognosis in severe COVID-19 and other systemic inflammatory states [13]. However, IL-6 was not measured in the present cohort, and the current findings therefore cannot determine whether inflammatory activity contributed to the observed differences between the intubation-timing groups.

Patients intubated within 24 hours had lower systolic blood pressure and higher serum lactate at admission. The median systolic blood pressure was 88.45 mmHg in the early-intubation group compared with 97.83 mmHg in the delayed-intubation group, while serum lactate was 3.03 mmol/L compared with 2.35 mmol/L, respectively. These findings are consistent with greater physiological and circulatory stress at presentation and may have contributed to the clinical decision to proceed with earlier intubation. They also indicate that early intubation was not confined to patients with a more favorable initial clinical profile. However, these variables alone do not establish greater overall disease severity or hemodynamic instability, and most other recorded admission variables did not differ significantly between groups. Systolic blood pressure and lactate were examined as group characteristics rather than as predictive markers, and their ability to identify HFNC failure or determine the appropriate time for intubation was not assessed.

The mortality finding requires cautious interpretation. No in-hospital deaths occurred among the 22 patients in the early-intubation group (0 of 22 patients, 0.0%), whereas 15 of 87 patients (17.2%) died in the delayed-intubation group. Firth penalized logistic regression was used because of the zero-event cell, and the adjusted confidence interval remained wide. The result supports an association between intubation timing and mortality within this cohort, but it does not establish that intubation within a fixed 24-hour interval prevents death. Disease trajectory, physician selection, unmeasured organ dysfunction, treatment limitations, and access to critical care resources may also have contributed to the observed difference.

Successful ventilator weaning was substantially more frequent in the early-intubation group. Earlier transition to invasive ventilation may have limited prolonged respiratory muscle loading before intubation, although this mechanism was not directly measured. Differences in underlying disease severity, complications, sedation, ventilator management, and response to treatment may also affect liberation from mechanical ventilation. The observed association should therefore be interpreted as a cohort-level finding rather than as evidence that the same timing strategy would produce equivalent results in every patient.

The shorter duration of the initial controlled mechanical ventilation phase and shorter ICU length of stay in the early-intubation group were consistent with the weaning findings. The longer overall hospital stay appears to move in the opposite direction. Death can shorten observed hospital length of stay, whereas patients who survive the acute phase may require prolonged ward care, rehabilitation, management of comorbidities, and additional time before discharge. Differences in post-ICU recovery requirements and local discharge practices may also have contributed to the observed difference. A survivor effect is therefore a plausible explanation for the longer hospitalization in the early-intubation group, but this interpretation was not tested using a survivor-only or competing-risk analysis.

Immediate ICU admission should therefore not be interpreted as an independent benefit of early intubation. Differences between ward-based and ICU-based care, including monitoring intensity, staffing, sedation practices, ventilator management, and weaning procedures, may also have influenced subsequent outcomes and cannot be separated from the effect of intubation timing in the present observational analysis. The finding also illustrates how resource availability can influence the apparent relationship between treatment timing and subsequent care.

The results support close monitoring of patients receiving HFNC, with repeated assessment of oxygenation, arterial blood gas findings, respiratory effort, hemodynamic status, and neurological condition. They do not support automatic intubation solely because 24 hours have elapsed. The clinical implication is that continued HFNC treatment should be reassessed when deterioration persists and that transition to invasive ventilation should not be postponed when the clinical picture indicates that non-invasive support is insufficient [17,9].

Limitations

This study has several limitations. First, its observational and non-randomized design does not permit causal interpretation of the associations between intubation timing and clinical outcomes. The decision to intubate was based on arterial blood gas findings, the patient's clinical course, and physician judgment, and unmeasured factors such as the cause of ARDS, respiratory effort trajectory, organ dysfunction, vasopressor requirements, treatment-limitation decisions, and differences in supportive care may have influenced both intubation timing and subsequent outcomes. Adjustment for the available baseline variables reduces but does not eliminate confounding by indication. The definition of the groups according to the interval from HFNC initiation to intubation also introduces a potential timing-related bias, since patients classified in the delayed-intubation group necessarily remained alive and without intubation for more than 24 hours. This limitation should be considered when interpreting the observed associations between intubation timing and subsequent outcomes. Furthermore, because the exposure groups were defined according to the elapsed time from HFNC initiation to intubation and the analysis was restricted to patients who ultimately underwent intubation, selection bias and timing-related or guarantee-time bias cannot be excluded. In addition, this was a single-center study conducted within a specific clinical pathway in which immediate ICU transfer depended on bed availability and some patients remained mechanically ventilated in medical wards before ICU admission. Differences in staffing, monitoring intensity, sedation practices, ventilator management, weaning procedures, and ICU resource availability may therefore have affected both treatment location and subsequent clinical outcomes.

The final analysis included 109 patients, with only 22 patients in the early-intubation group, and mortality events were sparse, including no deaths among patients intubated within 24 hours. Although Firth-penalized logistic regression was used to address complete separation, the resulting estimates remained imprecise, as reflected by the wide confidence intervals, and should therefore be considered exploratory. HFNC failure was defined operationally as the subsequent requirement for endotracheal intubation and invasive mechanical ventilation, without a separate prospectively standardized physiological failure time point or single threshold determining escalation. The ROX index was not prospectively recorded or used to guide escalation; respiratory deterioration was instead assessed clinically using oxygenation, arterial blood gas findings, respiratory distress, and accessory respiratory muscle use. Consequently, the findings do not establish an optimal intubation threshold or support intubation after a fixed duration of HFNC therapy. Furthermore, the final analysis included only patients who ultimately required invasive mechanical ventilation, excluding those who responded successfully to HFNC, and the results therefore should not be generalized to all patients receiving HFNC therapy. Standardized critical care severity scores (including APACHE II, SAPS II, and SOFA), detailed ventilator mechanics (e.g., positive end-expiratory pressure (PEEP) and plateau pressure), serial measures of respiratory effort, and long-term outcomes (e.g., 30-day or 90-day mortality), functional status, and health-related quality of life were not available. Future multicenter studies should also consider serial inflammatory and prognostic biomarkers, including IL-6 where clinically appropriate, alongside physiological measures to better characterize the trajectory of HFNC failure and its relationship with the timing of escalation.

## Conclusions

In this prospective observational cohort of patients with mild-to-moderate ARDS who ultimately required invasive mechanical ventilation after HFNC therapy, intubation within 24 hours of HFNC initiation was associated with lower observed in-hospital mortality. This mortality estimate was imprecise, with a wide 95% confidence interval, and should therefore be interpreted cautiously. Earlier intubation was also associated with more frequent successful ventilator weaning, a shorter ICU stay, and a shorter initial controlled mechanical ventilation phase. Patients intubated within 24 hours had a longer overall hospital stay, a finding that may partly reflect differences in survival and subsequent care requirements.

These findings identify an association between earlier escalation to invasive mechanical ventilation and several more favorable clinical outcomes among patients in whom HFNC ultimately failed. They support close clinical monitoring during HFNC therapy and timely escalation when worsening oxygenation, arterial blood gas deterioration, increased respiratory effort, hemodynamic instability, or neurological decline indicate that non-invasive support is insufficient. However, the observational design and restriction of the analysis to patients who ultimately underwent intubation introduce the potential for selection bias and confounding by indication. In addition, classification according to the interval between HFNC initiation and intubation may be susceptible to timing-related or guarantee-time bias. The limited sample size, sparse mortality events, residual confounding, and center-specific intubation and ICU admission practices further limit causal interpretation and generalizability. Larger multicenter studies using standardized assessment of HFNC failure, prospectively defined escalation criteria, and appropriate time-dependent analytical approaches are needed to confirm these associations and better define the clinical significance of intubation timing.

## References

[1] Ranieri VM, Rubenfeld GD, Thompson BT (2012). Acute respiratory distress syndrome: the Berlin definition. JAMA.

[2] Matthay MA, Thompson BT, Ware LB (2021). The Berlin definition of acute respiratory distress syndrome: should patients receiving high-flow nasal oxygen be included?. Lancet Respir Med.

[3] Matthay MA, Arabi Y, Arroliga AC (2024). A new global definition of acute respiratory distress syndrome. Am J Respir Crit Care Med.

[4] Bellani G, Laffey JG, Pham T (2016). Epidemiology, patterns of care, and mortality for patients with acute respiratory distress syndrome in intensive care units in 50 countries. JAMA.

[5] Mauri T, Turrini C, Eronia N, Grasselli G, Volta CA, Bellani G, Pesenti A (2017). Physiologic effects of high-flow nasal cannula in acute hypoxemic respiratory failure. Am J Respir Crit Care Med.

[6] Dysart K, Miller TL, Wolfson MR, Shaffer TH (2009). Research in high flow therapy: mechanisms of action. Respir Med.

[7] Frat JP, Thille AW, Mercat A (2015). High-flow oxygen through nasal cannula in acute hypoxemic respiratory failure. N Engl J Med.

[8] Oczkowski S, Ergan B, Bos L (2022). ERS clinical practice guidelines: high-flow nasal cannula in acute respiratory failure. Eur Respir J.

[9] Grasselli G, Calfee CS, Camporota L (2023). ESICM guidelines on acute respiratory distress syndrome: definition, phenotyping and respiratory support strategies. Intensive Care Med.

[10] Koulenti D, Tsigou E, Rello J (2017). Nosocomial pneumonia in 27 ICUs in Europe: perspectives from the EU-VAP/CAP study. Eur J Clin Microbiol Infect Dis.

[11] Brochard L, Slutsky A, Pesenti A (2017). Mechanical ventilation to minimize progression of lung injury in acute respiratory failure. Am J Respir Crit Care Med.

[12] Yoshida T, Amato MB, Kavanagh BP, Fujino Y (2019). Impact of spontaneous breathing during mechanical ventilation in acute respiratory distress syndrome. Curr Opin Crit Care.

[13] Niculet E, Chioncel V, Elisei AM (2021). Multifactorial expression of IL-6 with update on COVID-19 and the therapeutic strategies of its blockade (review). Exp Ther Med.

[14] Kang BJ, Koh Y, Lim CM (2015). Failure of high-flow nasal cannula therapy may delay intubation and increase mortality. Intensive Care Med.

[15] Xixi NA, Kremmydas P, Xourgia E, Giannopoulou V, Sarri K, Siempos II (2022). Association between timing of intubation and clinical outcomes of critically ill patients: a meta-analysis. J Crit Care.

[16] Yasuda H, Okano H, Mayumi T, Nakane M, Shime N (2021). Association of noninvasive respiratory support with mortality and intubation rates in acute respiratory failure: a systematic review and network meta-analysis. J Intensive Care.

[17] Lee KG, Roca O, Casey JD (2024). When to intubate in acute hypoxaemic respiratory failure? Options and opportunities for evidence-informed decision making in the intensive care unit. Lancet Respir Med.

[18] von Elm E, Altman DG, Egger M, Pocock SJ, Gøtzsche PC, Vandenbroucke JP (2007). The Strengthening the Reporting of Observational Studies in Epidemiology (STROBE) statement: guidelines for reporting observational studies. Lancet.

[19] Firth D (1993). Bias reduction of maximum likelihood estimates. Biometrika.

[20] Heinze G, Schemper M (2002). A solution to the problem of separation in logistic regression. Stat Med.

[21] Lun CT, Leung CK, Shum HP, So SO (2022). Predictive factors for high-flow nasal cannula failure in acute hypoxemic respiratory failure in an intensive care unit. Lung India.

[22] Miller DC, Pu J, Kukafka D, Bime C (2022). Failure of high flow nasal cannula and subsequent intubation is associated with increased mortality as compared to failure of non-invasive ventilation and mechanical ventilation alone: a real-world retrospective analysis. J Intensive Care Med.

